# Guideline for RSA and CT-RSA implant migration measurements: an update of standardizations and recommendations

**DOI:** 10.2340/17453674.2024.40709

**Published:** 2024-05-30

**Authors:** Bart L KAPTEIN, Bart PIJLS, Lennard KOSTER, Johan KÄRRHOLM, Maury HULL, Abby NIESEN, Petra HEESTERBEEK, Stuart CALLARY, Matthew TEETER, Trevor GASCOYNE, Stephan M RÖHRL, Gunnar FLIVIK, Laura BRAGONZONI, Elise LAENDE, Olof SANDBERG, L Bogdan SOLOMON, Rob NELISSEN, Maiken STILLING, the International Radiostereometry Society ()

**Affiliations:** 1 Department of Orthopedics, Leiden University Medical Center, Leiden, The Netherlands; 2 Department of Orthopedics, Sahlgrenska University Hospital, Gothenburg, Sweden; 3 Orthopedic Surgery Department, University of California, Davis, United States; 4 Orthopedic Research Department, Sint Maartenskliniek, Nijmegen, The Netherlands; 5 Department of Orthopedics and Trauma, Royal Adelaide Hospital, Adelaide, Australia; 6 Department of Medical Biophysics, Western University, London, Canada; 7 Orthopedic Innovation Centre, Winnipeg, Canada; 8 Division of Orthopaedic Surgery, Oslo University Hospital, Oslo, Norway; 9 Department of Orthopedics, Skane University Hospital, Lund, Sweden; 10 Life Quality Studies, University of Bologna, Bologna, Italy; 11 Department of Surgery, Dalhousie University, Halifax, Canada; 12 Sectra, Linköping, Sweden; 13 Department of Orthopedics, Aarhus University Hospital, Aarhus, Denmark

## Abstract

**Opening remarks** — These guidelines are the result of discussions within a diverse group of RSA researchers. They were approved in December 2023 by the board and selected members of the International Radiostereometry Society to update the guidelines by Valstar et al. [1]. By adhering to these guidelines, RSA studies will become more transparent and consistent in execution, presentation, reporting, and interpretation. Both authors and reviewers of scientific papers using RSA may use these guidelines, summarized in the Checklist, as a reference. Deviations from these guidelines should have the underlying rationale stated.

Since its introduction in 1974 [2], radiostereometric analysis (RSA) has been used and is a valuable tool to assess migration of joint replacements (prostheses) with sub-millimeter and sub-degree accuracy [3,4]. The high accuracy of RSA allows for relatively small study groups to obtain clinically relevant results. Migration of joint replacements is an early phase of loosening, making measurements of implant migration an important tool to assess one important aspect of their performance: prosthesis–bone fixation [5-7]. Migration analysis is important for the evaluation and phased introduction of new prosthesis designs and surgical techniques as it provides an early warning for aseptic loosening, and therefore potentially prevents the introduction and continuation of inferior prostheses [5,6,8-13]. In addition, RSA is also considered the most accurate method to measure hip and knee prosthesis liner wear in vivo [14-18], to study skeletal growth [19,20], fracture healing [21-23] and other fields of research, such as endovascular stent migration [24].

In 2005, guidelines for the standardization of RSA of implants were published to facilitate consistency in the execution, presentation, and interpretation of RSA migration studies [1]. In 2013, the ISO standard on RSA of implants was published [25]. Since then, knowledge and experience in clinical RSA studies have increased.

Migration assessment methods have been further developed and introduced such as model-based RSA, line-emitted scanning radiography (EOS)-based migration measurements, and computed tomography (CT)-based migration measurements (CT-RSA) [26-34). Recent developments in CT-RSA have shown that CT-RSA has high potential to become an alternative to the classical marker-based RSA as it seems to have similar accuracy and it does not require markers or a calibration box. The main limitation of CT-RSA is the higher radiation dose [35]. In addition, artificial intelligence (AI), which is a very useful tool in image analysis [36], is rapidly developing to measure prosthesis migration in both CT [37] and standard clinical radiographs [38]. While there seems to be an increasing role of AI in orthopedic image measurements as well as to analyze these measurements [39-42], it is increasingly important to unbox the black box of AI so that AI-based conclusions are explainable to doctors and patients [43,44].

**Table:** CHECKLIST. This reporting checklist is intended to serve as a checklist table specifically for prosthesis migration studies as an addition to the CONSORT [107] or STROBE [108] guidelines or any other guideline depending on study type. The items presented here should be viewed as the minimum. Authors are encouraged to provide additional information when deemed necessary.

Section/topic	Checklist item	Page reported
**Title and abstract**		
Identification	Identification as a radiostereometric analysis (RSA) study or CT-based radiostereometric analysis (CT-RSA) study in the title.	
	Identification as a radiostereometric analysis (RSA) study or CT-based radiostereometric analysis (CT-RSA) study in the abstract and keywords.	
**Methods**		
Study details	Report papers/references where prior results or partial results can be found (e.g., the 2-year results have been previously reported [REF]).	
	First and last inclusion (e.g., March 1998–December 2000).	
	Country and hospital(s) where surgeries were performed.	
	Number of surgeons (and number of surgeries per surgeon) that performed the surgeries	
Study groups	Detailed description of prosthesis, cement/coating, and liner characteristics for each study group.	
Follow-up	Report whether the first postoperative examination was obtained before or after weightbearing.	
	Mean number and SD of days between surgery and the baseline RSA examination.	
	Mean number and SD of days between surgery and the primary endpoint RSA examination.	
RSA technique	Migration measurement method (marker-based RSA, model-based RSA, CT-RSA).	
	Patient position (supine, weightbearing).	
	Software used, including version number.	
	Location and orientation of the migration coordinate system.	
	Use of fictive/feature points to calculate MTPM.	
Marker-/model-based RSA technique		
	Image resolution (DPI) and type (CR, DR, film) of X-ray detectors.	
	Material and size of markers.	
	Calibration cage used, including type (uniplanar, bi-planar).	
	Cut-off values for condition number and mean error of rigid-body fitting.	
	Consistent- or all-marker method for RSA analysis.	
CT-RSA technique	CT-scanner brand and model.	
	Voxel size, slice thickness, kV, mAs.	
	Was metal artifact reduction used.	
	Effective radiation dose in mSv (for hip, spine, shoulder).	
**Results**		
Study flow	Number of migration examinations for each study group and follow-up timepoint used in the primary analysis.	
	Number and reasons why migration examinations (including double examinations) were missing or excluded; may also be reported in the methods.	
Outcome	All migration data should be presented in millimeters (translations) and degrees (rotations).	
	Double examinations: mean, SD, and n for all outcome variables in the study (including 3 translations, 3 rotations, MTPM, TT, and TR if relevant) should be presented in a table for each study group separately.	
	Mean and SD of number of markers, condition number, and mean error of rigid-body fitting for each rigid body (bone/prosthesis) at the primary follow-up timepoint.	
	Unmodelled (raw data) of translation, rotation, and MTPM results: mean, n, and one of the following [CI, SD], or median and interquartile range for non-normal data for each study group and follow-up timepoint should be presented in a table or figure or both. If this table or figure does not fit in the manuscript, then it should be placed in supplementary data, or at least be available upon request.	
Revision/failures	Number of prosthesis revision/failures in each treatment group, including reason (e.g., revision due to aseptic loosening).	
	Migration values at last follow-up before revision or failure.	

Abbreviations: RSA: radiostereometric analysis, CT: computed tomography, DPI: dots per inch, CR: computed radiography, DR: digital radiography, mAs: milliAmpere-seconds, mSv: milliSievert, SD: standard deviation, n: number (of measurements), MTPM: maximum total point motion, CI: 95% confidence interval.

Some studies have suggested that improvements to the 2005 RSA guidelines are required to ensure better adherence in the future to common standards [45,46]. For these reasons, an update on the guidelines for standardization of implant migration measurements is required, including recommendations for CT-RSA.

## Terminology

Radiostereometric analysis (preferred medical subject headings [MeSH] term), radiostereometry, and roentgen stereophotogrammetric analysis are synonyms and their common abbreviation is RSA. RSA can be further categorized as marker-based RSA, using only markers [2], model-based RSA, using shape matching of a 3-dimensional (3D) model to define the position and orientation of the prosthesis or bone [26], and marker-free RSA without using any markers [32].

We suggest the use of the name CT-RSA for measuring migration by means of computed tomography. CT-RSA brings both new possibilities and challenges to prostheses migration measurements. It differs from standard RSA with both strengths and weaknesses, which it is worthwhile to elucidate [35]. As the body of knowledge for CT-RSA is growing rapidly, this method will be addressed in this paper as well.

Migration is defined as prosthesis displacement over time, whereas inducible displacement is defined as displacements occurring instantaneously (reversible or irreversible) as a result of an external load such as weightbearing [47-51]. Further terminology on migration measurements is provided in the relevant sections of this paper.

## Markers

**Figure 1:**
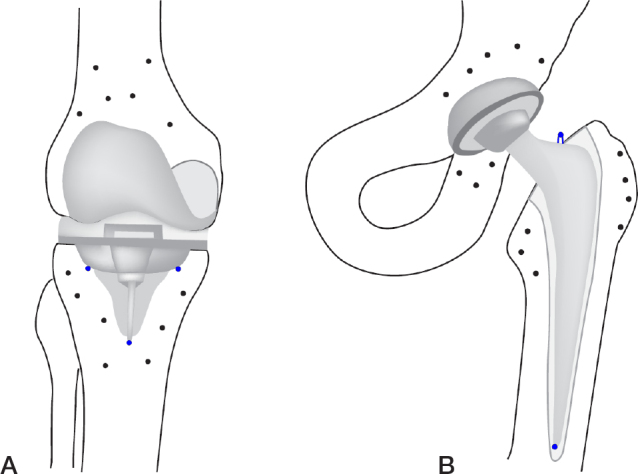
Knee (A) and hip (B) prosthesis with bone markers and prosthesis markers (blue) on the tibial component of the knee and femoral component of the hip.

In RSA, spherical tantalum markers, typically 0.5, 0.8, or 1 mm in diameter, are used to provide well-defined reference points [52,53]. The markers are inserted into the bone around the prosthesis intraoperatively. At least 3 non-collinear markers are necessary in each rigid body (e.g., prosthesis and bone) to measure translations and rotations. Markers can be occluded (e.g., by the metal prosthesis), end up outside the field of image, or become unstable over time. To ensure the precision and sustainability of the markers, it is therefore advised to insert 5–8 markers into each bone, close to and preferably well dispersed in 3 dimensions around the prosthesis (Figure 1). For marker-based RSA, markers are attached to the prosthesis, or inserted into the polyethylene component of the prosthesis. When placing markers in a polyethylene component intraoperatively, it is strongly recommended to use a guiding device to place the markers at predetermined optimized locations [54,55], which are in the non-weightbearing zones of the liner. Because of complexities in marker placement, the number of attached prosthesis markers is usually kept to a minimum (in most instances 3 markers). However, for reasons of increased demands on certification, implant manufacturers do not attach markers to the prostheses any longer. For model-based RSA, prosthesis markers are not necessary [26,56,57], and for CT-RSA, neither bone nor prosthesis markers are necessary [30,35].

The stability and distribution of markers within a rigid body influences the accuracy of the migration calculation. The mean error of rigid-body fitting (ME) is commonly used to assess the stability of markers over time. It is the root mean square of the differences in positions between the reference markers in the baseline examination and their matching markers in the follow-up examination after applying the rigid-body transformation that aligns these follow-up markers to the reference markers [25,58,59]. We recommend 0.35 mm to be the upper limit for ME [1].

The distribution of markers relative to collinearity can be assessed using the condition number (CN) [25,60]. High CNs indicate poor marker distributions, while low CNs indicate appropriate marker distribution [60]. Based on long experience from the Swedish RSA research groups, we recommend that the upper limit for the CN for total hip arthroplasty (THA), total knee arthroplasty (TKA), unicondylar knee arthroplasty (UKA), and total shoulder arthroplasty (TSA) studies is 120 mm^–1^ [1]. For smaller joints, the anatomy limits the distribution of markers, making use of higher CNs necessary [61]. In these cases, and as a general rule, it is essential to validate the precision of the measurements using double examinations.

For RSA, either a consistent set of markers throughout subsequent RSA examinations (“consistent-marker method”) or all available markers at each follow-up that can be matched to the baseline RSA image (“all-marker method”) can be used. At the group level, there seems to be no significant difference, but at the individual patient level, the consistent-marker method provides more consistent results [62]. Using the consistent marker method may alter migration results at earlier timepoints when markers become unstable or occluded at later timepoints and newer analysis may use fewer markers for migration calculation, changing the results with respect to the earlier analysis (e.g., 5 years’ follow-up after 2 years’ follow-up publication).

To avoid missing data as a result of CN and ME thresholds, as well as the marker selection method, appropriate solutions should be used such as the marker-configuration model [63]. In some cases, higher CNs may also be accepted in combination with sufficient (4 or more) stable markers (low ME). We suggest performing statistical analysis on the data meeting the ME/CN criteria as stated in the protocol (reduced/more concise data set), and in addition, a second analysis using all available data including CNs above the criterion (complete data set). Differences between the 2 analysis methods should be discussed.

In RSA, the ME and CN are quality measures that provide internal validation of the measurements. A current shortcoming of CT-RSA is that generally accepted quality measures currently do not exist [35]; although first proposals have been described in the literature to quantify quality of CT image registration [64], they require more extensive studies.

## Radiographic setup

**Figure 2:**
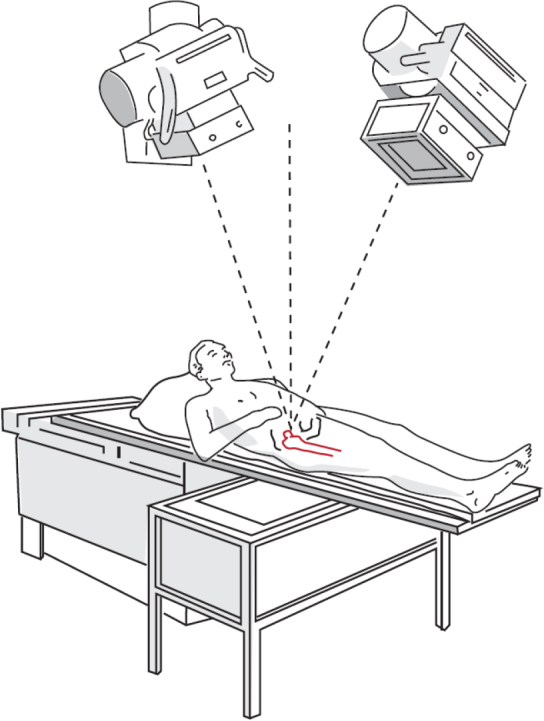
RSA uniplanar setup consisting of a calibration cage underneath the prosthesis of interest and 2 roentgen tubes angled at a 40° angle to each other. The roentgen detectors are placed in a slot below the calibration cage.

The radiographic setup for model-based/marker-based RSA consists of the calibration cage (or calibration box) in combination with the X-ray equipment. The calibration cage contains markers at known positions and is used to define the global coordinate system, calibrate the roentgen images, and calculate the positions of the 2 roentgen foci. For hips and shoulders, a uniplanar setup is most commonly used, meaning that the 2 roentgen detectors are positioned side-by-side with an approximately 40° angle between the roentgen beams [65-67] (Figure 2). For knees and most other joints in the extremities, either a uniplanar or biplanar setup is used. In a biplanar setup, the 2 roentgen detectors are, most commonly, placed at a 90° angle relative to each other. Apart from the radiographic setup, it is also important to describe the detector type (digital radiography [DR], computed radiography [CR], or scanned analogue roentgen films) [68]. For all systems, the spatial- and grayscale resolution are related to the precision of the analysis. A minimum of 150 dots per inch (DPI) and a minimum grayscale resolution of 8 bits is recommended [69]. New or alternative radiographic setups need to be properly validated before being used in clinical migration studies [70].

RSA radiographs are usually made with non-conventional (i.e., non-anatomic) projection directions and roentgen settings (high voltage [V] and low milliampere-seconds [mAs]). Therefore, RSA radiographs are typically not used for diagnostic purposes. In general, the effective radiation dose of an RSA radiograph is lower than that of a standard radiograph [68,71,72]. Due to the low radiation dose, RSA studies usually fall in “Category I: Effective doses less than 0.1 millisievert (mSv) (adults)” according to European Union (EU) regulations. This level of risk is considered to be trivial [73].

CT-RSA does not require a calibration cage. A slice thickness < 1.0 mm is recommended [35], as is a pixel size < 0.5 mm and a scan protocol using metal artefact reduction. For CT-RSA it is important to provide information regarding the CT scanner brand and model (cone beam or regular CT), scan protocol, voxel size, slice thickness, kVp (V) exposure (mAs), and whether metal artifact reduction was employed. While the method is compatible with lower-than-usual CT-radiation doses, the resultant effective dose is non-trivial [73]; an account of the radiation dose for each CT-RSA study is therefore important.

## Coordinate systems

**Figure 3:**
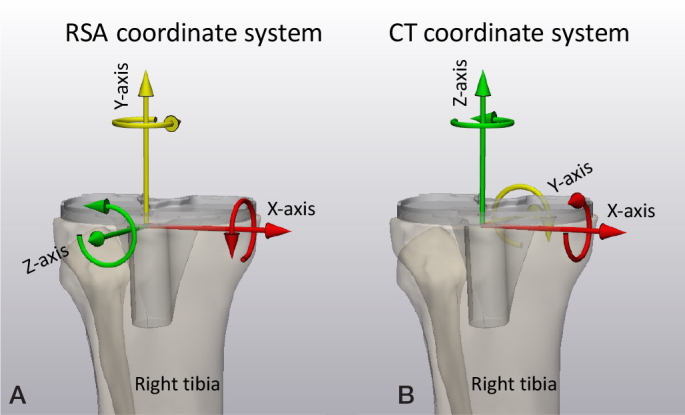
(A) The RSA migration coordinate system for a right-sided tibia with X-axis pointing medially, Y-axis pointing superiorly, and Z-axis pointing anteriorly. (B) The CT migration coordinate system for right-sided tibia with X-axis pointing medially, Y-axis pointing posteriorly, and Z-axis pointing superiorly.

For RSA, the global coordinate system is defined by the calibration cage. Usually, in standard patient positioning, the X-axis points to the left side of the patient, the Y-axis is superior, and the Z-axis anterior. For CT-RSA the standard Digital Imaging and Communication in Medicine (DICOM) coordinate system is used with the X-axis pointing to the left side of the patient, Y-axis to posteriorly, and the Z-axis superiorly (Left–Posterior–Superior, LPS). Note that the CT-RSA coordinate system is different from the RSA coordinate system (Figure 3).

**Figure 4:**
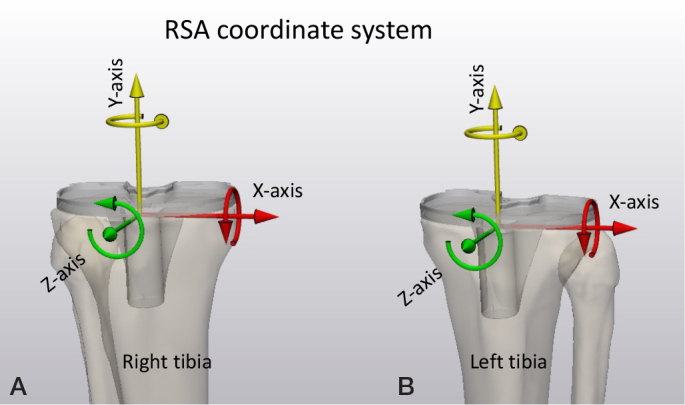
The RSA migration coordinate system for a right-sided (A) and a left-sided (B) tibia. For left-sided tibias, translations in the X-axis direction, and rotations about the Y-axis and Z-axis must be multiplied by minus one to convert them to right-side tibias. For the CT coordinate system (shown in Figure 3B), translations in the X-axis direction, and rotations about the Y-axis and Z-axis must be multiplied by minus one to convert them to right-sided tibias.

To describe prosthesis migration, one option is to use a migration coordinate system that is aligned with the anatomy of the patient. The default RSA migration coordinate system is always based on migration of implants in the right-hand side of the patient. The X-axis points medially, the Y-axis superiorly, and the Z-axis anteriorly (Figure 4). The origin of the migration coordinate system is defined in the baseline examination and should be positioned at a location in the migrating rigid body (prosthesis) that facilitates interpretation of translations. It is important that the origin and orientation of the migration coordinate system remains the same within a patient for all follow-up moments. Ideally, it should also be the same between different patients. The geometrical center of the rigid body is commonly used as origin [2]. Note that by having the patient aligned with the calibration cage, the migration coordinate system is also aligned with the global coordinate system for RSA. For CT-RSA, the patient is aligned with the CT scanner, thus the default migration coordinate system for CT-RSA is aligned with the DICOM LPS coordinate system. To avoid confusion, an anatomic description of the coordinate system, such as medial, superior, anterior, should be included and used to describe migration in all clinical papers. Migration results of left-sided prostheses have to be converted into the right side before results can be aggregated into group results (Figure 4).

A non-anatomic alignment of the rigid body (prosthesis) of interest to the global coordinate system is an alternative approach that might be advantageous to visualize markers [74], reduce the registration error [75], obtain migration results with higher clinical relevance, and/or reduce noise caused by kinematic cross-talk [76-78]. It is then required that an adjusted position and orientation of the migration coordinate system be used and described.

## Migration

Implant migration is measured over time relative to a baseline examination made postoperatively. It is calculated in 2 steps: first, the reference rigid body (bone) in the follow-up examination is aligned with the corresponding reference rigid body in the baseline examination by applying a transformation. This removes the difference in patient position and orientation between baseline and follow-up. The same transformation is applied to the migrating rigid body (prosthesis) in the follow-up examination. The remaining difference in position and orientation of the migrating rigid body between the baseline and transformed follow-up examination represents migration [78]. Ideally, the position of the patient should be as uniform as possible between examinations to minimize errors [79,80].

To calculate migration, for example from 1-year follow-up to 2-year follow-up, for each patient, the migration at the 1-year follow-up is subtracted from the migration at 2-year follow-up, as opposed to recalculating migration at 2-year follow-up using the 1-year follow-up as baseline examination.

## Rigid-body motion

There are different methods to quantify migration. The most informative method is rigid-body motion describing the motion of the whole rigid body (prosthesis) using 3 translations and 3 Euler rotations. Rigid-body motion is fully dependent on the position and orientation of the migration coordinate system. The 3 translations are the translations of the origin of the migrating coordinate system along the axes of the migrating coordinate system, and the Euler rotations are about the axes of the migrating coordinate system in body-fixed XYZ sequence [2].

Rigid-body translations can be summarized by a single value, total translation (TT), corresponding to the length of the translation vector, which can be calculated using the Pythagorean theorem. The same applies to rigid-body rotations, which can be presented as total rotation (TR). Note that the Pythagorean theorem can only be used to summarize small (i.e., < 5°) rotations [2].

## Point motion

Point motion describes the translation of specific points on the prosthesis such as the tip of the hip stem in THA migration studies [81,82] or the medial and lateral points on tibial base-plates for TKA migration studies [83,84]. These points can be added as fictive points, or feature points, to the rigid bodies in the analysis software. Similar to rigid-body motion, the point motion can be summarized by its vector length.

## Maximum total point motion (MTPM)

MTPM is defined as the length of the translation vector corresponding to the point on the prosthesis that has moved most, disregarding its location. For model-based RSA, the large number of points on the outer surface ensures that the maximum motion is always captured. If the number of available points on the implant is limited, such as with the use of fictive points and/or marker-based RSA, MTPM refers to the fictive point/marker that has moved most [85]. Note that the use of a consistent set of fictive points is advised for marker-based RSA and CT-RSA in order to create a consistent set of points to calculate MTPM, especially at an individual patient level [62]. MTPM is the most frequently used metric to present TKA migration. A great advantage of MTPM to present motion is that it is not dependent on the migration coordinate system being used and that it is a summary measure. It makes no assumptions of the migration failure mechanisms, and it is very sensitive for picking up migration because it is a summary measure. Note that with MTPM, the point of measurement may not only differ between patients, but also within the same patient over time. Other drawbacks of MTPM, being an unsigned value such as TT and TR, is that the direction of motion is unknown, and it is a biased metric as mean MTPM increases with increased measurement errors and larger implant size [85].

Be aware that to assess the average direction of prosthesis migration in a group of patients, motions in opposite directions for individual patients might result in the mean motion being zero while there is significant motion in each individual patient [86]. For this reason, it is recommended to use signed values for translations and rotations, including values of the data scatter, and to also include MTPM, and/or TT, TR, or the vector length of point motion, in cases where absolute values are required.

## Accuracy

The high accuracy of RSA is the main reason why small-scale studies can be performed. Accuracy is defined by trueness and precision, also known as bias or systematic error and random error respectively. Trueness is the closeness of agreement between the mean value obtained from a large series of test results and an accepted reference (the “true” or “gold standard”) value. Precision is the closeness of agreement between independent test results obtained under stipulated conditions [87,88]. When introducing a new RSA method or equipment, it is important to determine the accuracy of the method in a phantom experiment. The phantom should be able to apply a translation and/or rotation in/about as many axes as possible, preferably X, Y, and Z to a migrating rigid body (prosthesis) relative to a reference rigid body (bone) with an accuracy that is at least an order of magnitude (10x) better than that of the assessed migration measurement method (a “gold standard” method). For this, different phantoms using micro-manipulators have been developed and used [17,18,32,67,89,90]. We recommend presenting the accuracy results as the mean (i.e., bias) and standard deviation (i.e., random error or precision) of the differences in signed translations and rotations as well as the unsigned MTPM, and/or TT and TR between the gold-standard and measured values. To compare 2 methods for calculating migration in the absence of a gold standard, the Bland–Altman method is recommended [91]. For studying specific aspects of migration accuracy, digital simulation studies can be a valuable tool [85,92].

In clinical studies, bias and random error can be assessed by so-called “double examinations.” Double examinations are 2 X-ray (RSA) examinations of the same patient that are made on the same day with the patient and equipment repositioned within limits that are expected to be encountered during a clinical follow-up study. In this short time interval, the prosthesis is not expected to have moved with respect to the reference bone. However, due to measurement errors, motion could be measured/calculated. Both bias and random error can be calculated by using one of the double examinations as a baseline examination and computation of the migration between the 2 examinations [93]. As the actual motion is assumed to be zero, “accuracy of zero motion” is also used to indicate the migration results of double examinations. When presenting the results of double examinations, a table containing the number of double examinations, the mean (i.e., bias) and standard deviation (i.e., random error or precision) of the migration calculations for all outcome variables, and for each group in the study (including 3 translations, 3 rotations, TT, TR, and MTPM if applicable) should be included. As double examinations are important for correct interpretation of the migration results, it is highly recommended to include double examinations for all study patients. In the case that only a limited number of double examinations is allowed by the medical ethics committee, this should be stated in the manuscript; a minimum of 25% of the study patients is recommended and selection bias should be avoided. A timepoint at 1-year follow-up or earlier is recommended to avoid patients being lost to follow-up for the double examinations. For CT-RSA, double exams are currently also recommended, although future studies should investigate that for CT-RSA double exams are not necessary, or can be replaced by intra-segmental migration results or another alternative [64].

## Clinical study: recommendations/practical issues

### Pre-clinical study

- A phantom experiment before starting a clinical study is recommended to optimize marker placement and estimate precision [26].

### Patient position

- Supine examinations for implant migration [79].
- Loaded examinations for TKA wear/kinematics [15,94].
- Supine examinations may be sufficient for THA wear [16].

### Baseline examination

- Immediately postoperatively, before weightbearing is preferred.
- In clinical practice, as early as possible, within 2 weeks postoperatively.
- All patients have the same loading protocol, i.e., before or after weightbearing.
- Specify mean and SD of days between surgery and baseline RSA examination, before or after weightbearing and before or after hospital discharge in the manuscript.
- In cases where the baseline examination was made longer than 2 weeks postoperatively, migration values are likely not comparable with migration values (including migration thresholds) from the literature using a baseline examination within 2 weeks postoperatively. This should be mentioned in the discussion as a study limitation and should be made explicitly clear in the figures (axis) and abstract as well.

### Follow-up examinations

- A minimum of 2 follow-up examinations to measure a migration pattern is required.
- A minimum of 2 years for implant migration studies.
- A minimum of 5 years for THA and TKA wear studies [14,95].
- Timepoints at 6 weeks, 3, 6, 12, and 24 months postoperatively (time-window: ±2 weeks for timepoints before 12 months, and ±10% for timepoints at 12 months and later).
- Timepoints at 5, 7, 10, 15, and 20 years for mid- and long-term studies.
- Present mean number and SD of days for the primary outcome (e.g., 24 months postoperatively).

### Number of patients

- Calculate the sample size for every clinical randomized controlled RSA trial (see Appendix 1).
- Adjust the sample size to compensate for possible dropouts, including RSA technical issues such as: poor bone-marking, poor image quality, markers being superimposed (occluded) by prostheses, etc.
- Analyze baseline examinations as soon as possible and exclude the patients who have unsuitable baseline examinations from further follow-up RSA examinations (secondary exclusion criteria) to prevent unnecessary radiation exposure for these patients. However, these patients should remain in the study for proper follow-up of other study parameters.
- In some studies, excluded patients are replaced by continuing patient inclusion until the desired number of patients with sufficient postoperative data quality is reached. Make sure to adjust the study protocol accordingly to prevent medical ethical issues concerning the number of patients included in the study.
- In the CONsolidated Standards of Reporting Trials (CONSORT) or Strengthening the Reporting of Observational Studies (STROBE) flow diagram, all missing migration results in the follow-up caused by technical issues should be mentioned in the analysis section of the diagram, as this is important to assess whether selection bias such as attrition bias may have occurred.

## Analysis of results

In general, studies of prosthesis migration include comparatively few patients who undergo repeated measurements. For these types of studies, it is advised to use suitable statistical analysis techniques such as (generalized) linear mixed models [96]. We recommend presenting migration results on a group level as mean and 95% confidence interval (CI) of the mean. For comparative studies, the migration difference (mean and CI) between the groups should also be presented.

## Interpretation of results

Migration, calculated by RSA, is a validated surrogate marker for long-term primary joint replacement outcomes in terms of aseptic loosening [97-100]. It can be applied to a wide range of purposes including early implant safety studies [5], screening, and diagnostics of implant loosening [101]. The application of RSA to different purposes requires that researchers, clinicians, clinical guideline developers, systematic reviewers, and patients be aware of the scenario that applies to a particular study purpose to interpret the results correctly.

## Implant safety studies

When RSA is used for implant–bone fixation safety studies, early migration at a group level (e.g., mean migration indices during early follow-up) is used to estimate the revision rate in the future for a particular implant, technique, or fixation method with corresponding confidence intervals. This estimate of revision is subsequently compared with a benchmark (e.g., mean revision rate in national joint registries) to determine whether the implant is likely to be more or less safe regarding the risk of revision for aseptic loosening. However, in reality, the RSA migration is compared with migration thresholds, which are used as substitutes for revision rates [11,98,102,103]. The emphasis on implant safety studies lies on safety and therefore strict thresholds are required. Since TKA and THA have reached the plateau phase in a so-called hype cycle, the risk of a new design is generally greater than the (promised) benefit [104]. Hence a strict approach seems justified to reduce the risk of introducing implants to the market that later turn out to be unsafe [5]. The potential disadvantage of a strict approach is that some safe implants could initially be classified as “at risk” or as “unsafe,” therefore delaying or halting their clinical use. However, the alternative of a less strict approach would increase the risk of initially classifying some unsafe implants as safe, which could potentially be a disaster for patients and could even undermine the credibility of research regarding implant safety. The latter stresses the importance of a national arthroplasty register alongside the early warning of implant migration studies. It should be stressed that implant safety studies provide information on a group level, not on individual patient level. To make statements on the number (or percentage) of patients (implants) with a particular classification, such as number of patients with continuous migration, “at risk” or “unacceptable” requires a high number of study patients to create statistically significant results [86], which is usually not the case in standard RSA studies.

## Implant screening studies

When RSA is used in the scenario of screening, the implant migration is measured in patients without symptoms or signs with the purpose of estimating the future risk of implant revision or clinical failure for that patient. Typically, the migration of the patients is classified, at a patient level, as continuous migration (e.g., more than a chosen threshold) or stable (less than a chosen threshold) during the second postoperative year [11]. Reaching a plateau phase after initial migration is more important than the time point of reaching the plateau phase. It is important to realize that the posterior (post-test) probability (risk of implant loosening) depends on the a priori probability (overall revision risk) and results of the test (classification as continuously migrating or not). This is elaborated in Supplementary data where it becomes apparent that the estimated 10-year risk of revision for 2 patients with different implant designs can be very different (17% versus 62%) while the RSA results (classification as continuously migrating) are the same, due to the differing overall revision rates for the 2 implant designs.

## Implant diagnostic studies

When RSA is used in the scenario of diagnostics, the migration or inducible displacement of implants is measured in patients with symptoms or radiological signs with the purpose of determining whether the implant is loose or not. Since the patient has symptoms or signs (e.g., pain on weightbearing and progressing radiolucent lines on radiographs), the a priori risk of loosening is much higher than in a screening scenario where patients do not have complaints. Inducible displacement holds great promise for diagnostics because it provides information on implant fixation from measurements from a single follow-up (e.g., during loading/standing) and is correlated to RSA-measured implant migration [48,105,106].

It is important to realize the differences between screening and diagnostics versus implant safety studies. Screening and diagnostic studies focus on the individual patient, in a clinical setting, and are mostly designed to diagnose—rule in—pathology, while implant safety studies focus on the implant, standardized surgical techniques, or method of fixation, which is a group-level factor, and are designed to ensure safety for patients by ruling out unsafe—disaster—implants with regard to implant stability.

## Funding and disclosures

Neither the International Radiostereometry Society nor any of the authors received funding for this paper. BK, PH, SC, TG, SR, RN, and MS are board members of the International Radiostereometry Society. OS is a full-time employee at a company selling CT-RSA software. Complete disclosure of interest forms according to ICMJE are available on the article page, doi: 10.2340/17453674.2024.40709

## Supplementary data

Example of positive predictive value for 2 TKAs with different a priori revision risks is available as supplementary data on the article page, doi: 10.2340/17453674.2024.40709

## Supplementary Material



## Figures and Tables

**Figure 5:**
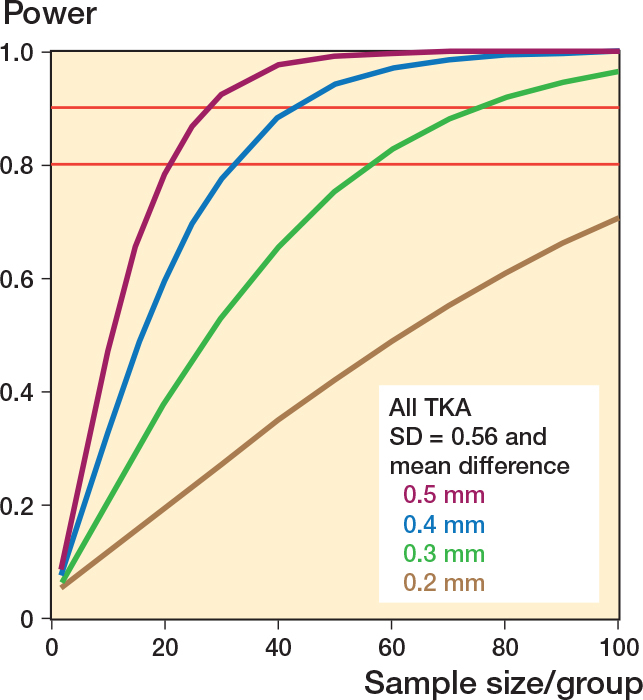
Relation between power and sample size for all TKAs with the standard deviation (SD = 0.56) of the expected outcome.

**Figure 6:**
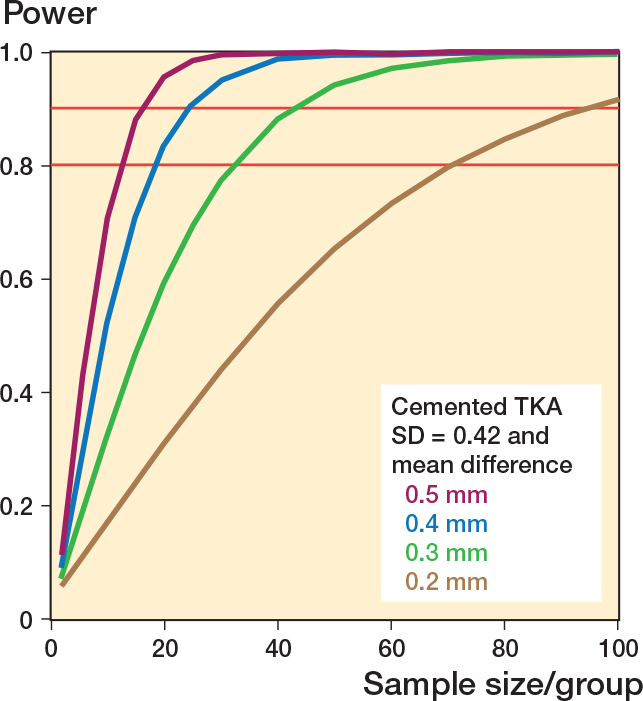
Relation between power and sample size for cemented TKAs with the standard deviation (SD = 0.42) of the expected outcome.

**Figure 7:**
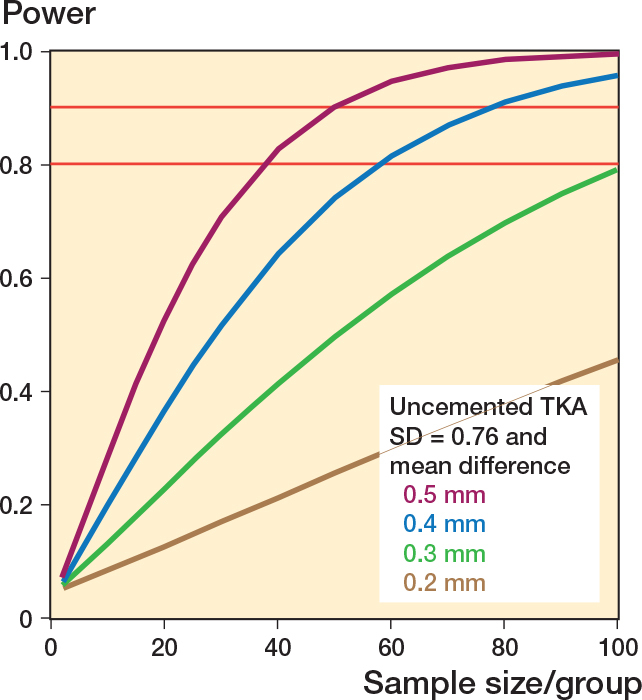
Relation between power and sample size for uncemented TKAs with the standard deviation (SD = 0.76) of the expected outcome.

## Notes

BK, BP, and LK wrote the first draft. All authors contributed to the design of the paper and processed it for critical content.

Handling co-editors: Keijo Mäkelä and Philippe Wagner

*Acta* thanks Hannu T Aro and Rami Madanat for help with peer review of this manuscript.

## Appendix

### Sample size calculations

An important aspect when designing RSA studies is a sample size calculation. For example: how many total knee arthroplasties are needed to detect a clinically meaningful difference in migration? Although advanced statistical methods such as generalized linear mixed models are often used in RSA studies [96], the statistical test on which the sample size calculation is based is often a standard independent samples t-test for the migration at final follow-up.

Figures 5–7 show the relation between power and sample size and can be used by researchers as a tool to help determine the sample size for an RSA study [109,110]. The graphs for Figures 5–7 were generated using the R package (R Foundation for Statistical Computing, Vienna, Austria) “pwr.”

Assuming that the standard deviations (SD) are different, the sample size is determined for each group/treatment arm. The lines represent an expected clinical meaningful mean difference between groups of 0.5 mm (purple), 0.4 mm (blue), 0.3 mm (green), and 0.2 mm (brown). SD is the standard deviation of the expected outcome. It is important to realize that the SD should also include the biological variation and hence it is not the same as the standard deviation obtained from the double examinations or a phantom experiment. As the SD is unknown at the start of the study, an estimation is made. In this example, the mean SD (MTPM at 1 year) from a systematic review was used [10], but in other studies it is advised to select an SD from historical data from a previously published study of similar prosthesis design. Figures 6 and 7 show the effect of different SDs for cemented and uncemented implants, respectively, on the sample size calculations. The red horizontal lines indicate the 80% and 90% power levels. The sample size calculations in these figures are based on mean MTPM at 1 year with alpha = 0.05.

Statistical methods that use the repeated measures design of RSA studies have more statistical power than the t-test that was used for the sample size calculations in Figures 5–7 and thus require a smaller sample size [111]. However, missing values (RSA examinations) decrease the power of a study and could occur for RSA studies due to technical issues, e.g., insufficient markers visible or patients who are lost to follow-up, miss a follow-up, or are revised during the study period. It is therefore advisable to increase the sample size by, e.g., 10% to account for possible missing values.
