# Supplementary material for: Guideline for RSA and CT-RSA implant migration measurements: an update of standardizations and recommendations
Source: Acta Orthop. 2024 May 30;95:256–67. doi: 10.2340/17453674.2024.40709 (PMC11141406; doi:10.2340/17453674.2024.40709)
Supplement: Supplementary file 1 [file ActaO-95-40709-s1.pdf]

Supplementary data

Example of positive predictive value for 2 TKAs with different a priori revision risks

In the study by Ryd et al. [1], the positive predictive value of the change in MTPM between 1 and 2 years (i.e. continuous migration) of more than 0.2 mm being predictive for early tibial component loosening was 42%, the sensitivity was 100%, the specificity was 84.7%, and the revision rate for 10 years follow-up was 9.9% (Table 1).

Table 1. Contingency matrix using data from the study of Ryd et al. (1).

		**Outcome** (revised at 10 years follow-up)		
		**Positive**	**Negative**	
**Predicted**<br>(MTPM 2–1 years > 0.2 mm)	**Positive**	**True positive**<br>(TP) = 13	**False positive**<br>(FP) = 18	31
	**Negative**	**False negative**<br>(FN) = 0	**True negative**<br>(TN) = 100	100
		13	118	131
Revision rate (10 years follow-up)		13/131	9.9%	
Sensitivity	TP/(TP+FN)	13/13	100%	
Specificity	TN/(FP+TN)	100/118	84.7%	
Positive predictive value	TP/(TP+FP)	13/31	42%	
Negative predictive value	TN/(FN+TN)	100/100	100%	
Positive likelihood ratio	sensitivity / (1 – specificity)		6.5	
Negative likelihood ratio	(1 – sensitivity) / specificity)		0.0	

Let us consider 2 patients with continuous migration of more than 0.2 mm. Patient A has received TKA "A" with a known revision rate of 3% at 10 years in a study of 500 patients, while patient B has received TKA "B", a disaster implant, with known revision rate of 20% at 10 years in a study of 500 patients. Given this information, and assuming the same sensitivity and specificity for RSA as calculated by Ryd et al. [1] (Table 1), what is the estimated risk of revision at 10 years for patient A and for patient B?

Table 2. Contingency matrix for patient A having a prosthesis with 3% aseptic loosening at 10 years

		**Outcome** (revised at 10 years follow-up)		
		**Positive**	**Negative**	
**Predicted**<br>(MTPM 2–1 years > 0.2 mm)	**Positive**	**True positive**<br>(TP) = 15	**False positive**<br>(FP) = 74	89
	**Negative**	**False negative**<br>(FN) = 0	**True negative**<br>(TN) = 411	411
		15	485	500
Revision rate (10 years follow-up)		15/500	3%	
Sensitivity	TP/(TP+FN)	15/(15)	100%	
Specificity	TN/(FP+TN)	411/(74+411)	84.7%	
Positive predictive value	TP/(TP+FP)	15/(15+74)	16.8%	
Negative predictive value	TN/(FN+TN)	411/411	100%	
Positive likelihood ratio	sensitivity / (1 – specificity)		6.5	
Negative likelihood ratio	(1 – sensitivity) / specificity		0.0	

Table 3. Contingency matrix for patient B having a prosthesis with 20% aseptic loosening at 10 years

		**Outcome** (revised at 10 years follow-up)		
		**Positive**	**Negative**	
**Predicted** (MTPM 2–1 years > 0.2 mm)	**Positive**	**True positive** (TP) = 100	**False positive** (FP) = 61	161
	**Negative**	**False negative** (FN) = 0	**True negative** (TN) = 339	339
		100	400	500
Revision rate (10 years follow-up)		100/500	20%	
Sensitivity	TP/(TP+FN)	100/(100)	100%	
Specificity	TN/(FP+TN)	339/(61+339)	84.7%	
Positive predictive value	TP/(TP+FP)	100/(100+61)	62%	
Negative predictive value	TN/(FN+TN)	339/339	100%	
Positive likelihood ratio	sensitivity / (1 – specificity)		6.5	
Negative likelihood ratio	(1 – sensitivity) / specificity		0.0	

These examples (Tables 2 and 3) demonstrate that the estimated 10-year risk of revision for patient A (16.8%) is different from the 10-year risk of revision for patient B (62%), when their implants have continuous migration more than 0.2 mm. The reason is that the 10-year risk of revision for each patient depends on the a-priori risk (pre-test risk) as well as the diagnostic accuracy of the test. Since the a-priori risk of 10-year revision is lower for patient A (3% revision rate) than for patient B (20% revision rate), the risk of revision after a positive test is also lower. The relation between a-priori risk (revision rate) and the risk of revision, the posterior probability, for a patient after a positive test (continuous migration > 0.2 mm) is depicted in Figure 1.

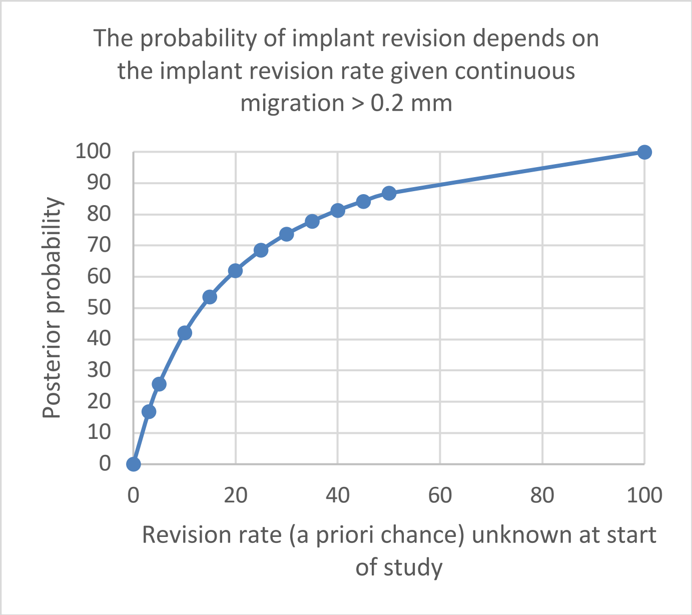

Figure 1. The relation between a-priori risk (revision rate) and posterior probability of revision for continuous migration more than 0.2 mm based on data from the study of Ryd et al. [1].

The reconstruction of contingency tables for every patient is not practical and the risk of revision, the posterior probability, changes when a-priori probability changes (Figure 1). Therefore, the positive or negative likelihood ratio may offer a practical solution: the post-test odds that the patient has (or will get) implant loosening is estimated by multiplying the pre-test odds by the likelihood ratio. The use of likelihood ratios requires the use of odds instead of risks, which may render the calculations a bit more complex. However, the use of Fagans Nomogram [2] or, more modern, the use of online calculators or apps can make these calculations a lot easier. For continuous migration > 0.2 mm, the positive likelihood ratio is 6.5 and the negative likelihood ratio is 0 [1].

Sometimes the proportion of patients with continuous migration is used in studies to evaluate the long-term revision rate and hence the results from such studies could be interpreted in the context of implant safety evaluation. For instance, studies may report that there were 10 out of 50 patients with continuous migration > 0.2 mm. The question now arises, how many of those 10 patients will be revised in the long term, in other words, what is the expected long-term revision rate. For this question, we go back to the example above on patient A with TKA "A" (3% revision rate) and patient B with TKA "B" (20% revision rate): for patients with TKA "A," 16.8% of 10 patients are expected to be revised in the long-term and for patients with TKA "B" this is 62% of 10 patients. Thus, the percentage of patients with continuous migration > 0.2 mm that are expected to be revised depends on the revision rate and can be different between different study groups. Additionally, in implant safety studies, the revision rate of an implant is unknown, so it is impossible to know how many patients are expected to be revised in the long-term. It is therefore not advisable to use the number of patients with continuous migration > 0.2 mm or "at risk" for implant safety studies.

For implant safety studies the interpretation should be based on the mean migration or derived metrics compared with contemporary thresholds or minimally clinically important differences.

The proportion of patients with continuous migration > 0.2 mm may be reported for each group, but this should not be used in the interpretation of implant (patient) safety as the a priori risk is unknown.

References

1. Ryd L, Albrektsson B E, Carlsson L, Dansgard F, Herberts P, Lindstrand A, et al. Roentgen stereophotogrammetric analysis as a predictor of mechanical loosening of knee prostheses. J Bone Joint Surg Br 1995; 77(3): 377-83. PMID: 7744919
2. Fagan T J. Nomogram for Bayes's Theorem. N Engl J Med 1975; 293(5): 257. doi: 10.1056/NEJM197507312930513.
